# Anoctamin1 Induces Hyperproliferation of HaCaT Keratinocytes and Triggers Imiquimod-Induced Psoriasis-Like Skin Injury in Mice

**DOI:** 10.3390/ijms22137145

**Published:** 2021-07-01

**Authors:** Mi Ran Choi, Hae Dong Kim, Sinyoung Cho, Seong Ho Jeon, Dong Hyun Kim, Jungwon Wee, Young Duk Yang

**Affiliations:** 1 Laboratory Animal Research Center, Ajou University School of Medicine 164, Worldcup-ro, Yeongtong-gu, Suwon 16499, Korea; mrchoi2007@ajou.ac.kr; 2 Department of Pharmacy, College of Pharmacy and Institute of Pharmaceutical Sciences, CHA University, 120 Haeryong-ro, Pocheon 11160, Korea; eanby12@nate.com (H.D.K.); jsy7122@naver.com (S.C.); cmb_jsh@naver.com (S.H.J.); 3 Department of Dermatology, CHA Bundang Medical Center, CHA University School of Medicine, 335 Pangyo-ro, Seongnam-si 13488, Korea; terios92@cha.ac.kr; 4 Department of Molecular Medicine and Biopharmaceutical Sciences, Graduate School of Convergence Science and Technology, Seoul National University, Gyunggy 16229, Korea; jungwona0727@snu.ac.kr

**Keywords:** anoctamin 1, ERK pathway, imiquimod, keratinocyte, psoriasis

## Abstract

Psoriasis, a long-lasting and multifactorial skin disease, is related to comorbidities such as metabolic disease, depression, and psoriatic arthritis. Psoriasis occurs due to a variety of factors including keratinocyte hyperproliferation, inflammation, and abnormal differentiation. Proinflammatory cytokines upregulated by increased activation of keratinocytes and immune cells in the skin trigger progression of psoriasis. This study aimed to investigate the effects of anoctamin1 (ANO1) on psoriasis development in vitro and in vivo. We analyzed the proliferation of HaCaT keratinocytes and ANO1-related ERK and AKT signaling pathways after ANO1 inhibitor (T16Ainh-A01 and Ani9) treatment and knock-down of *ANO1*. Furthermore, after applying imiquimod (IMQ) cream or coapplying IMQ cream and T16Ainh-A01 on mouse ears, we not only observed psoriatic symptoms, including ear thickening, but also quantified the effects of treatment on ERK and AKT signaling-involved proteins and proinflammatory cytokines. Inhibition of ANO1 attenuated the proliferation of HaCaT cells and induced reduction of pERK1/2. Coapplication of IMQ and T16Ainh-A01 on ears of mice reduced not only symptoms of IMQ-induced psoriasis such as thickening and erythema, but also expression of ANO1 and pERK1/2 compared to that of application of IMQ alone. In addition, the expression levels of *IL-17A*, *IL-17F*, *IL-22*, *IL-23*, *IL-6*, *IL-1β*, and *TNF-α* increased after applying IMQ and were significantly reduced by coapplying IMQ and T16Ainh-A01. These results aid in understanding the underlying mechanisms of ANO1 in epidermal layer keratinocyte hyperproliferation and suggest the potential of ANO1 as a target to treat psoriasis.

## 1. Introduction

Psoriasis is a chronic inflammatory skin disease characterized by clinical features such as erythematous plaques, papules, and pruritus and affects about 2% of the population worldwide, with a high degree of morbidity [1]. Patients with psoriasis often are reluctant to reveal the affected skin and can experience suicidal thoughts if they have comorbidities such as anxiety and depression [2]. The pathophysiology of psoriasis is multifactorial and is accompanied by hyperplasia of the epidermis through abnormal proliferation and differentiation of keratinocytes and inflammation with immunologic alterations in the skin [3,4,5]. Previous studies have reported that several cytokines, including IL-17, IL-22, IL-23, and tumor necrosis factor-α (TNF-α), are expressed highly in the epidermis of patients with psoriasis or the imiquimod-induced mouse model [6,7,8]. The increase and activation of keratinocytes and autoreactive skin T-cells stimulate upregulation of these cytokines, leading to a proinflammatory state [9,10,11]. Although a wide variety of research has been performed to prevent epidermal hyperplasia through inhibition of keratinocyte proliferation or management of the immune system based on such findings, there are no remarkable results. 

Anoctamin1 (ANO1), also known as transmembrane protein 16A (TMEM16A), is a chloride channel that is opened by intracellular calcium and membrane potential [12,13,14]. ANO1 is expressed widely in numerous cell types such as smooth muscle cells, epithelial and endothelial cells, interstitial cells of Cajal, and sensory neurons [14,15,16,17]. Among the 10 members of the anoctamin family, ANO1 has been studied most intensively in various physiological functions such as saliva and lacrimal secretion, smooth muscle contraction in blood vessels [18], pain perception in nociceptive neurons [19,20], and cell growth [14]. In particular, the expression of ANO1 was upregulated in various human cancers including breast cancer [21], esophageal cancer [22], and head and neck cancer [23]. In some cancer types, such as glioma, head and neck squamous cell carcinoma (HNSCC), colorectal cancer, and prostate carcinoma, inhibition of ANO1 promoted not only reduction of tumor size and cell proliferation [24], but also decrease in migration, invasion, and metastasis [25,26,27]. With regard to the underlying molecular mechanism of tumorigenesis induced by ANO1, previous studies identified ANO1 to promote development of breast cancer [21], growth of colorectal cancer [28], and cell proliferation in benign prostatic hyperplasia (BPH) [29] through an increase in phosphorylated ERK1/2 or AKT. In addition, upregulation of ANO1 was identified in asthma [30] and hypertension [31]. These observations imply that regulation of ANO1 is an important drug target for cancer and chronic diseases.

To date, many ANO1 inhibitors have been reported. Although classical CaCC blockers such as niflumic acid, flufenamic acid, 5-nitro-2-(3-phenylpropylamino)-benzoic acid (NPPB), and 4,4-diisothiocyano-2,2-stilbenedisulfonic acid (DIDS) effectively block ANO1 channel activity [14,32,33], these inhibitors have low potency and selectivity. Among the recently discovered ANO1 blockers, MONNA, Ani9, CaCCinh-A01, T16Ainh-A01, tannic acid, idebenone, luteolin, and Aa3 showed the highest potency and selectivity for ANO1 and inhibited cell proliferation in various cancer cell lines [34,35,36]. Therefore, studies on regulation of ANO1 by these inhibitors to quantify the proliferation of cells related to diseases including psoriasis as well as cancer following ANO1 inhibition would provide important insight into the mechanisms underlying disease prognosis and development of therapeutic solutions. 

On the basis of these findings, we hypothesized that ANO1 induces abnormal proliferation of keratinocytes, followed by psoriasis progression. Identification of the effects of ANO1 on abnormal proliferation of keratinocytes, one of the main causes of psoriasis progression, will help illustrate the underlying mechanisms that induce psoriasis progression and develop drugs for treatment of psoriasis. The HaCaT cell line is a spontaneously immortalized human keratinocyte cell line that has been broadly used for skin disease-related studies [37,38]. In this study, we investigated whether pharmacological inhibition and knock-down of ANO1 affected the proliferation of HaCaT cells and ANO1-related ERK and AKT signaling pathways. In addition, we distinguished the effects of ANO1 on psoriasis development and investigated ANO-related mechanisms underlying psoriasis development by inducing psoriasis-like symptoms in mouse ears using imiquimod (IMQ) cream.

## 2. Results

### 2.1. Expression of ANO1 in HaCaT Cells and Skin of Psoriatic Patient

**Figure 1:**
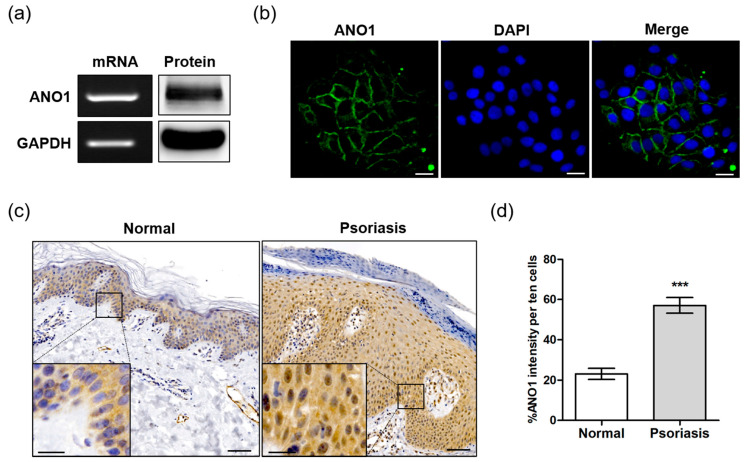
Expression of ANO1 in the human keratinocyte HaCaT cell line and psoriatic tissue. (**a**) The expression of ANO1 mRNA and protein levels in HaCaT cells. (**b**) Expression of ANO1 protein in HaCaT cells using immunocytochemistry. Scale bar = 50 µm. (**c**) The expression of ANO1 protein in psoriatic skin tissue was increased compared to that of healthy skin tissue. Scale bar = 50 µm (insert: 20 µm). (**d**) Expression of ANO1 in the skin of normal and psoriasis from immunohistochemical analysis (ANO1 intensity of ten cells per unit area was measured using ZEN 3.1). To compare the differences of ANO1 expression between normal and psoriasis skin tissues, Student’s *t*-test was used. *: significantly different from normal skin (*** *p* < 0.001).

To determine the role of ANO1 in psoriasis, we first investigated whether ANO1 was expressed in the HaCaT keratinocyte cell line or in human skin tissue. ANO1 mRNA and protein were highly expressed in HaCaT cells (Figure 1a). Based on immunocytochemical analysis, we observed that ANO1 was localized to cell membranes, confirming that ANO1 is a membrane-bound channel protein (Figure 1b). One of the typical hallmarks of psoriasis is sustained inflammation that leads to abnormal keratinocyte proliferation and dysfunctional differentiation [39]. To confirm the correlation between this abnormal proliferation and ANO1, we measured the expression levels of ANO1 mRNA and protein in human healthy and psoriatic lesion biopsies using qRT-PCR and immunohistochemical analysis. The expression of *ANO1* mRNA was increased (*p* = 0.059, Student’s *t*-test) in psoriasis tissues compared to healthy tissues (Figure S1). From the immunohistochemical staining results, typical acanthosis (epidermal hyperplasia) was observed in psoriatic skin, and ANO1 was upregulated throughout the epidermis of psoriatic lesions more than in normal skin (Figure 1c). Moreover, the expression levels of ANO1 protein in psoriasis tissues were also significantly higher than in normal tissues (Figure 1d). Therefore, considering keratinocytes are the most common cell type in the epidermis, it is believed that patients with psoriasis have a thicker epidermis composed of keratinocytes highly expressing ANO1 compared to normal subjects.

### 2.2. Effects of ANO1 on HaCaT Cell Proliferation and ERK and AKT Pathways

**Figure 2:**
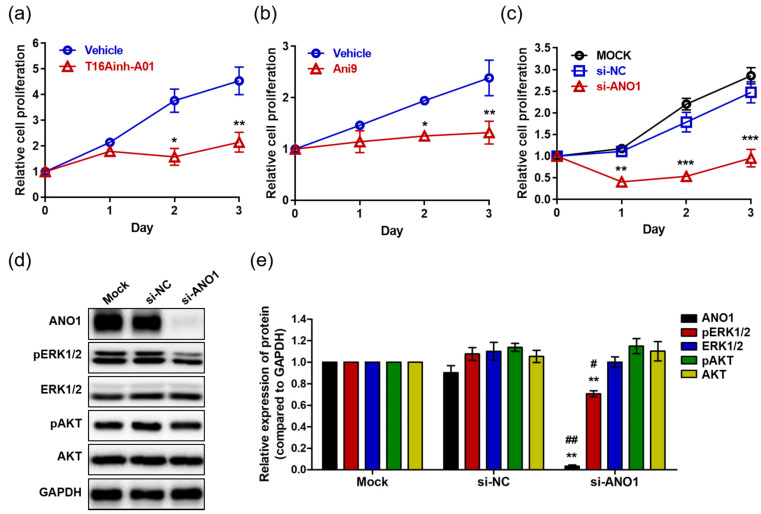
The effects of ANO1 inhibition on HaCaT cells. (**a**) Proliferation of cells exposed to 30 µM T16Ainh-A01 for 3 days. (**b**) Proliferation of cells exposed to 10 µM Ani9 for 3 days. (**c**) Proliferation of cells following *ANO1* knock-down for 3 days. After knock-down of *ANO1* using siRNA targeting *ANO1* and in negative controls (mock and si-NC), proliferation rates of HaCaT cells were measured for 3 days. (**d**) Immunoblots of ANO1 and ERK and AKT signaling pathway-related proteins after *ANO1* knock-down for 2 days. (**e**) Densitometric analysis for the relative intensity of ANO1 and ERK and AKT signaling pathway-related proteins. Treatments with the two blockers (T16Ainh-A01 and Ani9) and *ANO1* knock-down were performed as four independent replicates to guarantee reliable results. To compare the differences between control (vehicle or Mock [si-NC]) and blocker-treated or *ANO1* knock-down cells, two-way ANOVA and Tukey’s post-hoc tests were used. *: significantly different from vehicle in (**a**,**b**), from Mock and si-NC (**c**), and from Mock (**e**) (* *p* < 0.05, ** *p* < 0.01, and *** *p* < 0.001). ^#^: significantly different from si-NC (**e**) (^#^ *p* < 0.05 and ^##^ *p* < 0.01).

Studies have reported that ANO1 regulates proliferation in various cells and carcinomas including interstitial cells [40], prostate carcinoma [26], breast cancer [21], and HNSCC [24]. Thus, we analyzed the effects of ANO1 on proliferation of HaCaT cells using the ANO1 blockers T16Ainh-A01 and Ani9. HaCaT cells exposed to T16Ainh-A01 (30 µM) showed no difference in proliferation compared to the control cells at one day after exposure (Figure 2a). However, the proliferation of T16Ainh-A01-treated cells was significantly lower than that of control cells at two and three days post-treatment. In addition, Ani9-treated cells (10 µM) exhibited significantly lower proliferation than control cells two and three days after treatment, showing patterns like those of cells in a resting state (Figure 2b). Taken together, these results suggest that blocking the ANO1 channel induces delayed proliferation of keratinocytes or arrests their proliferation.

Considering that off-target toxicity is one of the most common side effects of pharmacological drugs, it is necessary to identify whether ANO1 is directly responsible for regulating keratinocyte proliferation. To do this, we induced knock-down of *ANO1* in HaCaT cells using siRNA targeting *ANO1* (si-ANO1). By observing proliferation rates for three days after knock-down of *ANO1*, we observed significant inhibition of cell proliferation from the first day to the third (Figure 2c). This result suggests that ANO1 directly mediates the proliferation of HaCaT cells. On the other hand, based on previous studies demonstrating that reduction of cell proliferation due to knock-down of *ANO1* is associated with phosphorylation of ERK or AKT [21,29], we evaluated the expression levels of ANO1 protein and proteins related to ERK and AKT signaling pathways two days after knock-down of *ANO1*. We found that the cells that were knocked down with si-ANO1 showed drastically decreased expression of ANO1 protein compared to negative control cells (Mock and si-NC) (Figure 2d). In addition, we observed that phosphorylation of ERK1/2 without change in expression levels was significantly reduced in cells that were knocked down by si-ANO1 (Figure 2e). However, knock-down of *ANO1* did not induce changes of expression or phosphorylation in AKT in the HaCaT cells. Therefore, knock-down of *ANO1* induces not only a reduction of proliferation, but also a decrease of pERK1/2 in HaCaT cells.

### 2.3. Direct Blocking of ANO1 Channel Activity by Resveratrol, Xanthohumol, Isoxanthohumol, and Curcumin

**Figure 3:**
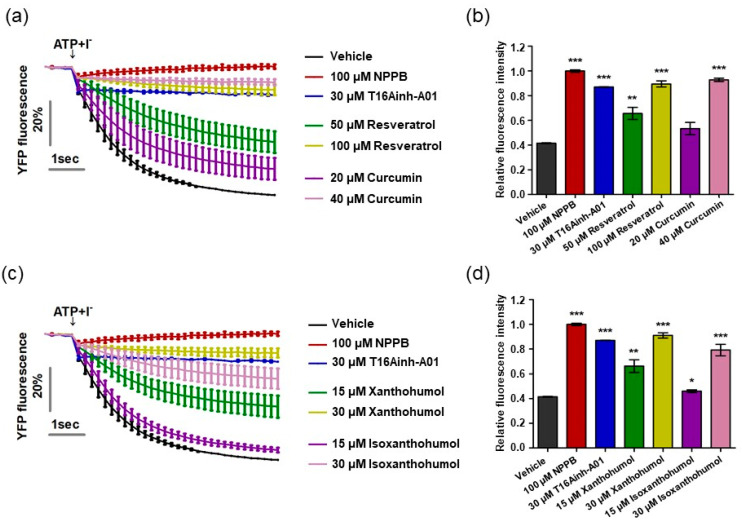
Inhibition of ANO1 channel activity by resveratrol, curcumin, xanthohumol, and isoxanthohumol. The activity of ANO1 was measured in Fischer rat thyroid (FRT) cells stably co-expressing human ANO1 and the yellow fluorescent protein (YFP). After the cells were exposed to positive controls (NPPB and T16Ainh-A01) or one of the four compounds for 20 min, they were treated with 140 mM NaI solution containing 100 µM ATP to activate ANO1 channel activity, continuously measured every 0.2 s for 6 s. (**a**) Fluorescence intensity of cells exposed to vehicle, positive controls, resveratrol, and curcumin. (**b**,**d**) Relative fluorescence intensity obtained from tested cells at 6 s. (**c**) Fluorescence intensity of cells exposed to vehicle, positive controls, xanthohumol, and isoxanthohumol. To compare the differences among vehicle, positive controls, resveratrol, curcumin, xanthohumol, and isoxanthohumol, two-way ANOVA and Tukey’s post-hoc tests were used. *: significantly different from vehicle (* *p* < 0.05, ** *p* < 0.01, and *** *p* < 0.001).

Previous research has reported that resveratrol, curcumin, xanthohumol, and iso-xanthohumol inhibit the hyperproliferation of keratinocytes in a psoriatic mouse model induced by IMQ cream [41,42,43]. Based on previous studies alongside our result demonstrating that ANO1 promotes hyperproliferation of keratinocytes, we predicted that inhibition of ANO1 activity by the four compounds would indicate its crucial role in the pathophysiology of psoriasis. On the basis of this prediction, we measured ANO1 channel activity using the halide-sensitive YFP imaging technique as described previously [34,44]. The halide-sensitive YFP imaging system is based on a cell line that presents reduced fluorescence intensity due to extracellular iodide influx induced by ANO1 activation. By measuring the inhibitory effects of resveratrol and curcumin on ANO1 activity in two dosages per compound for 6 s, the cells exposed to both 50 µM resveratrol and 20 µM curcumin showed a decrease in inhibitory effects on ANO1 as time went on, while the cells exposed to twice the concentration of each compound (100 µM resveratrol and 40 µM curcumin) maintained constant inhibitory effects on ANO1 for 6 s, as with positive controls (100 µM NPPB, a chloride channel inhibitor, and 30 µM T16Ainh-A01) (Figure 3a). The fluorescence intensity of cells exposed to 100 µM resveratrol and 40 µM curcumin at 6 s was similar to that of positive controls but significantly different from that of negative control (vehicle) (Figure 3b). The cells exposed to 30 µM xanthohumol and 30 µM isoxanthohumol also maintained constant inhibitory effects on ANO1 for 6 s, showing similar fluorescence intensity to the positive controls (Figure 3c,d). Therefore, since these four compounds directly inhibit activity of the ANO1 channel, it is supposed that ANO1 may be related to psoriasis.

### 2.4. Alleviation of Psoriasis Symptoms in Mice with Psoriatic Ears by Inhibiting ANO1 Activity

**Figure 4:**
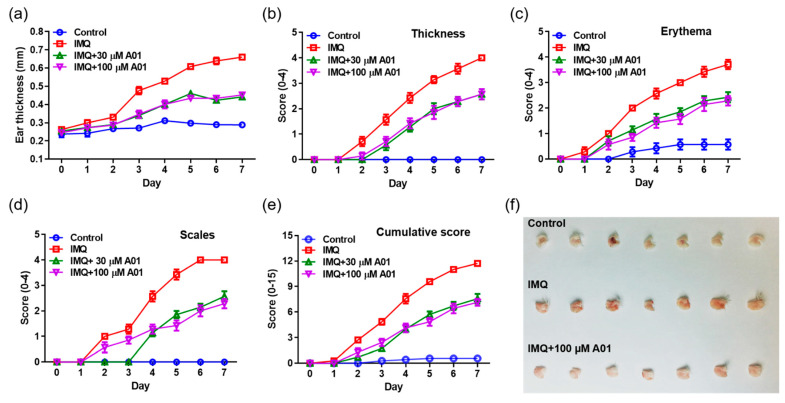
Alleviation of psoriasis-like symptoms in mouse ears by inhibiting ANO1. To induce psoriasis-like skin injury, three mouse groups (IMQ [*n* = 7], IMQ + 30 µM A01 [*n* = 7], and IMQ + 100 µM A01 [*n* = 7]) received a daily dose of 25 mg of 5% IMQ cream on their right ears for 7 consecutive days; IMQ + 30 µM A01 and IMQ + 100 µM A01 groups additionally received 15 µL of 30 µM and 100 µM A01, respectively, at 30 min before and after IMQ treatment. The control group received a similar dose of Vaseline cream on their right ears. (**a**) Changes in right ear thickness caused by IMQ and A01. (**b**–**d**) Thickness, erythema, and scaling of right ears were scored daily. (**e**) The cumulative score (thickening plus erythema plus scaling) was described. Symbols per day depict mean ± SEM of seven mice ears per group. The significant differences among groups obtained from statistical analysis are presented in Figure S2. (**f**) Phenotypic representation of the right ears in mice treated with IMQ or IMQ + 100 µM A01 for 7 days. Imiquimod, IMQ; T16Ainh-A01, A01.

To investigate whether pharmacological inhibition of ANO1 attenuates psoriasis-like symptoms in vivo, we applied IMQ cream and T16Ainh-A01, an ANO1 blocker, alone or together on the right ears of mice for seven consecutive days. We measured other typical psoriasis-like symptoms (erythema and scaling) as well as ear thickening every day from the day after the first application of the cream. As presented in Figure 4a, the ears of all IMQ-treated groups (IMQ, IMQ + 30 µM A01, and IMQ + 100 µM A01 groups) thickened over time. In particular, ear thickness of the IMQ group was increased drastically following IMQ application. On the other hand, ear thickness of the groups (IMQ + 30 µM A01 and IMQ + 100 µM A01 groups) that applied IMQ and T16Ainh-A01 together was increased significantly less compared to the group treated with IMQ alone (Figure S2a). Two days after the start of IMQ application, symptoms of thickening, erythema, and scaling started to appear (Figure 4b–d). In particular, these symptoms in IMQ group increased drastically over time (Figure S2b–d). Meanwhile, the groups that applied IMQ and T16Ainh-A01 together showed significantly slower increase in these symptoms compared to the IMQ group. The total scores of these symptoms for all groups are presented in Figure 4e and Figure S2e. Figure 4f shows right ears after sacrificing the mice on the eighth day. These results imply that application of IMQ and T16Ainh-A01 together weakens typical psoriasis-like symptoms.

### 2.5. Expression of ANO1 and ANO1-Related Proteins in Psoriatic Ears of Mice

**Figure 5:**
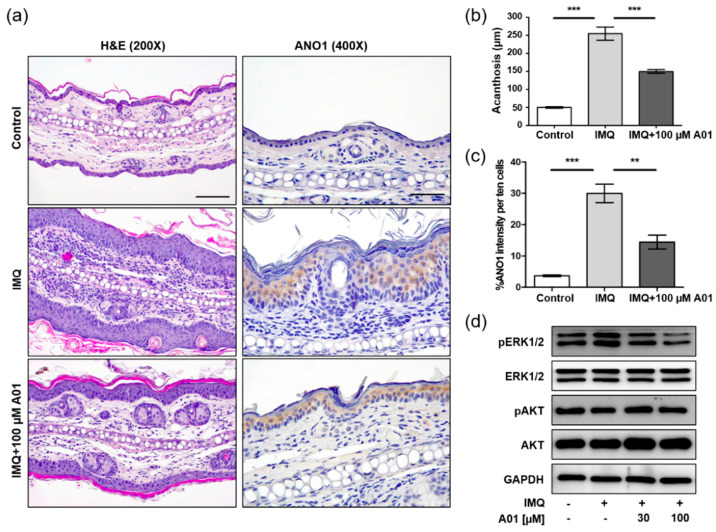
Changes in histopathological structure and ANO1-related proteins in mouse ears showing psoriasis-like symptoms caused by inhibiting ANO1. The right ears of the mice in all four groups (control, IMQ, IMQ + 30 µM A01, and IMQ + 100 µM A01 groups) were obtained on the eighth day after psoriasis-like skin injury and inhibition of ANO1. (**a**) H&E staining and ANO1 immunoreaction in the right ears of control, IMQ, and IMQ + 100 µM A01 groups. Scale bar = 500 µm. (**b**) The quantification of acanthosis in the right ears of control (*n* = 3), IMQ (*n* = 3), and IMQ + 100 µM A01 (*n* = 3) groups. (**c**) The signal intensity of ANO1 per ten cells in the epidermis of right ears of control (*n* = 3), IMQ (*n* = 3), and IMQ + 100 µM A01 (*n* = 3) groups. Significant differences of acanthosis (**b**) or ANO1 intensity (**c**) among control, IMQ, and IMQ + 100 µM A01 groups were analyzed using one-way ANOVA followed by Tukey’s multiple comparison test. *: significantly different from IMQ group (** *p* < 0.01 and *** *p* < 0.001). (**d**) The expression levels of ERK and AKT proteins. The phosphorylation of ERK was increased by IMQ cream but was decreased by A01 in a dose-dependent manner. Imiquimod, IMQ; T16Ainh-A01, A01.

We investigated histopathological changes in right ears of control, IMQ, and IMQ + 100 µM A01 groups on the eighth day using H&E staining. As the ears were thickest in the IMQ group, epidermal layers consisting of keratinocytes were thickest (Figure 5a) and the increase of acanthosis was also the highest in the IMQ group (Figure 5b). Furthermore, the IMQ group showed an increase in immune-related cells of the dermal layers. On the contrary, the IMQ + 100 µM A01 group exhibited thinner epidermal layers compared to the IMQ group. When we investigated ANO1 expression in the ears of three groups, the highest level of ANO1 was detected in the epidermal layer of those in the IMQ group (Figure 5a,c). These results suggest that IMQ induces hyperproliferation of keratinocytes that highly express ANO1, followed by thicker epidermal layers. Moreover, T16Ainh-A01 leads to reduction in IMQ-induced keratinocyte hyperproliferation and ANO1 expression.

Based on the observation that knock-down of *ANO1* induced reduction of pERK1/2 in HaCaT cells, we analyzed the expression levels of proteins related to ERK and AKT signaling pathways in the tested ears. The levels of pERK1/2 in the IMQ group were increased compared to the control group, but the levels in the IMQ + 30 µM A01 group and the 100 µM A01 group were decreased compared to the IMQ group (Figure 5d). These results suggest that the pivotal role of ANO1 in psoriasis is to affect the regulation of ERK phosphorylation.

### 2.6. Expression of Proinflammatory Cytokines in Psoriatic Ears of Mice

**Figure 6:**
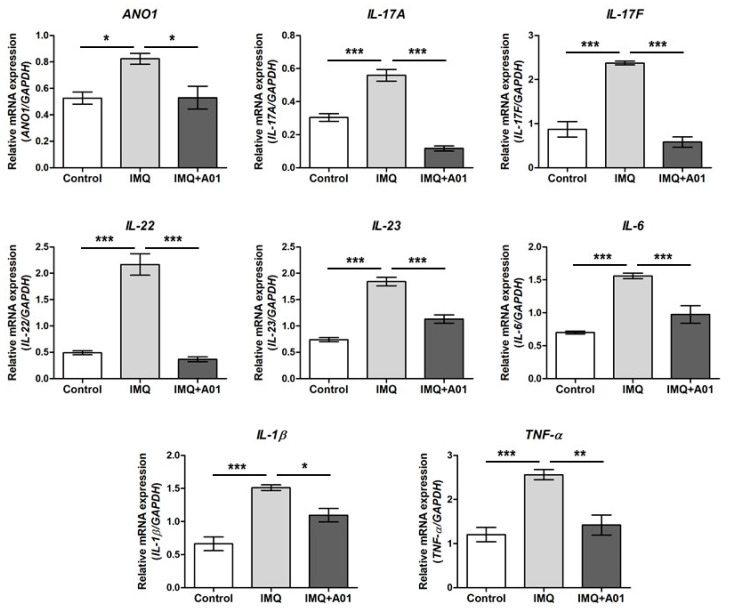
Changes in *ANO1* and immune response-related cytokine mRNA levels in mouse ears showing psoriasis-like symptoms caused by inhibiting ANO1. Two mouse groups (IMQ [*n* = 7] and IMQ + A01 [*n* = 7]) received a daily dose of 25 mg of 5% IMQ cream on right ears for 7 consecutive days, and the IMQ + A01 group additionally received 15 µL of 100 µM A01 at 30 min before and after IMQ treatment. The control group received a similar dose of Vaseline cream on their right ears. After extracting total RNA from the right ears of the mice (7 animals in each group), obtained on the eighth-day post-treatment onset, target genes were analyzed using RT-PCR.; their expression levels were measured and represented as densitometric graphs. To compare the differences among groups, one-way ANOVA and Tukey’s post-hoc tests were used. *: significantly different between two groups (* *p* < 0.05, ** *p* < 0.01, and *** *p* < 0.001). Imiquimod, IMQ; T16Ainh-A01, A01.

Assuming that psoriasis promotes expression of various proinflammatory cytokines including IL-23 and IL-17 [45,46], we investigated the gene expression of cytokines and *ANO1* in the right ears of control, IMQ, and IMQ + 100 µM A01 groups on the eighth day after application. The expression levels of *ANO1*, *IL-17A*, *IL-17F*, *IL-22*, *IL-23*, *IL-6*, *IL-1β*, and *TNF-α* in all mouse ears of the IMQ group were significantly higher than those of the control group (Figure 6). Interestingly, the expression levels of all genes in the IMQ + 100 µM A01 group were reduced similarly to those in the control group, showing a significant difference between IMQ and IMQ + 100 µM A01 groups. These results suggest that application of ANO1 blocker induces reduction of proinflammatory cytokines as well as ANO1 in psoriatic tissues.

## 3. Discussion

Hyperproliferation of keratinocytes and activation of T-cells in the epidermis stimulate the expression of cytokines, such as TNF-α, IL-17, and IL-23, followed by a proinflammatory state in the skin and finally psoriasis [6,9]. ANO1 is upregulated in a variety of cancers including glioma, colorectal cancer, and HNSCC, but its inhibition has led to decrease in tumor size, cell proliferation, and migration [23,24,25]. Based on previous findings, we investigated whether ANO1 was associated with proliferation of keratinocytes and inflammation of the epidermis or development of psoriasis-like symptoms using an animal disease model.

In the present study, we demonstrated that HaCaT cells, spontaneously immortalized human keratinocytes, highly express ANO1 mRNA and protein. In particular, ANO1 protein was expressed stably in the HaCaT cell membranes. HaCaT cells have been broadly tested in the verification of new compounds for psoriasis treatment and the study of molecular mechanisms related to psoriasis [47,48,49,50]. After discovering stable ANO1 expression in HaCaT cells, in addition to other studies, the HaCaT cell line is likely an accurate in vitro model to investigate ANO1-related mechanisms in keratinocyte hyperproliferation. In addition, we found that psoriasis tissue exhibited not only a thicker epidermis layer, but also stronger expression of ANO1 than normal tissue. These findings indicate ANO1-related mechanisms in HaCaT cells and animal models as evidence for the effect of ANO1 on psoriasis progression.

In this research, T16Ainh-A01 and Ani9 attenuated the proliferation of HaCaT cells for three days. As described in previous studies [51,52], T16Ainh-A01 and Ani9 are ANO1-specific channel inhibitors. In addition, ANO1 upregulated in cancer cells such as pancreatic cancer, lung cancer, and gastrointestinal stromal tumor cells was reduced by T16Ainh-A01 [53,54,55]. These results imply not only a positive correlation of ANO1 with poor prognosis in these cancers, but also the promising use of T16Ainh-A01 as a potential therapy for ANO1-overexpressing cancers. Furthermore, our observation that Ani9 inhibited proliferation of HaCaT cells is in agreement with another report that Ani9 inhibited the increased growth of cysts in mice with autosomal dominant polycystic kidney disease by upregulation of ANO1 [56]. As a consequence, Ani9 attenuates an increase in ANO1 in some disease progression-related cell proliferation. Based on these results, these ANO1 inhibitors are good candidates to study keratinocyte hyperproliferation-related psoriasis. Furthermore, in the present study, knock-down of *ANO1* caused a reduction in keratinocyte proliferation, confirming that ANO1 plays an important role in keratinocyte proliferation. 

Recently, some research groups have studied whether expression changes of ANO1 in several cancers and other disease models are responsible for alterations of AKT and ERK signaling. *ANO1* knock-down in breast cancer and colorectal cancer cells induced reduction of pERK1/2 and pAKT but not of ERK1/2 or AKT expression [21,28]. On the other hand, knock-down of *ANO1* upregulated in BPH promoted a decrease in only pAKT, implying that ANO1 affects the pathogenesis of BPH through the AKT signaling pathway [29]. Contrary to the findings identified in BPH, we observed that, in HaCaT cells, *ANO1* knock-down caused only a decrease in pERK1/2. Our results are consistent with those reported by Duvvuri et al. [24] in that *ANO1* knock-down in HNSCC both in vitro and in vivo led to an increase in pERK1/2 but no change in pAKT. Considering that *ANO1* knock-down affects the phosphorylation of both ERK and AKT in some diseases but phosphorylation of only one of the two proteins in other diseases, it is supposed that ANO1 participates in cell proliferation, migration, and disease progression through the AKT, ERK, or both pathways depending on cell type. Taken together, our results suggest that ANO1 enhances the proliferation of keratinocytes through the ERK pathway.

Previous studies have reported that IMQ induces psoriasis-like dermatitis, showing important phenotypes of psoriasis including thickening and erythema, and provokes inflammatory lesions in vivo [47,57,58,59]. In accordance with the results found in previous studies, our study induced psoriasis-like dermatitis on the ears of mice by applying IMQ cream. In particular, one of the hallmark psoriasis symptoms, thickening, was observed from two days after applying IMQ cream, showing a faster increase of thickening over time in comparison to the ears of the control group. On the contrary, applying T16Ainh-A01 drastically attenuated the thickening of treated ears due to IMQ at both 30 and 100 µM. In addition, the IMQ-treated group exhibited the most severe erythema, and the IMQ + T16Ainh-A01-treated group showed the mildest compared to the IMQ-treated group. Taken together, these results suggest that ANO1 inhibition alleviates the typical symptoms of psoriasis, thickening and erythema. 

Many research teams have sought to develop or investigate potential therapeutic candidates for psoriasis treatment. For example, the pharmacological effects of daphnetin [47], catalpol [59], and rice crude extract, consisting of anthocyanin [60], diosgenin [50], and botulinum toxin [57], have been evaluated after inducing psoriasis-like injury in animal models using IMQ. Some of these substances not only attenuated HaCaT cell proliferation, but also improved psoriatic injury in vivo, similar to our results of ANO1 inhibitor treatment. Although investigators continue to develop psoriasis treatments, no medicine is outstandingly effective as a psoriasis treatment without side effects. Moreover, even if some substances have been reported to have an effect on psoriasis, their pathophysiological and molecular mechanisms remain to be fully elucidated. On the other hand, we showed that IMQ-treated animals showed an increase in pERK1/2 protein that was reduced following ANO1 inhibitor treatment. Therefore, also considering the decrease in HaCaT pERK1/2 by *ANO1* knock-down, ANO1 likely plays a pivotal role in the progress of psoriasis-like dermatitis through the ERK pathway.

Skin is composed of various cell types; keratinocytes are the main cell type in the epidermis, followed by immune cells, including dendritic cells (DCs), macrophages, and T-cells, primarily composing the dermis [61]. These cell types modulate immune responses in the skin following stimulation. Microbiomes and other stressors activate keratinocytes, and the activated keratinocytes initiate psoriasis by triggering activation of plasmacytoid DCs (PDCs) that produce IFN-α [6]. When psoriasis persists or worsens, IFN-α and VEGF released by activated PDCs and keratinocytes provoke myeloid DCs (MDCs) that express proinflammatory cytokines including TNF-α, IL-1β, IL-6, and IL-23 [6]. In turn, the cytokines secreted from MDCs stimulate T-helper (Th) 17 and Th22 cells, which secrete IL-17A, IL-17F, and IL-22 [6]. Increased secretion of these cytokines is pathophysiological evidence of psoriasis. Assuming that immune response-related cytokines are increased in psoriasis, and that IMQ provokes inflammatory lesions through expression of cytokines, such as IL-6, TNF-α, IL-17A, and IL-23A, in vivo [47,57,59], we observed the expression levels of IL-17A, IL-17F, IL-22, IL-23, IL-6, IL-1β, and TNF-α mRNA and confirmed their increases following IMQ treatment, in agreement with the results reported in previous studies. Furthermore, IMQ treatment induced an increase of immune cells in the dermal layers. On the contrary, coapplication of IMQ and T16Ainh-A01 lowered the expression levels of cytokines and proliferation of immune cells to similar to those of the control group. These results suggest that ANO1, highly expressed in epidermal keratinocytes affected by psoriasis, modulates upregulation of cytokines as an upstream regulator through activation of immune cells in dermal layers.

In conclusion, we determined that ANO1 was expressed highly in HaCaT keratinocytes and the epidermal layer of patients with psoriasis. Based on these findings, we investigated the effects of ANO1 on HaCaT cell proliferation and psoriasis development in mouse skin. We demonstrated that inhibition of ANO1 attenuated the proliferation of HaCaT cells and reduced phosphorylation of ERK1/2, implying that ANO1 promotes hyperproliferation of keratinocytes through the ERK signaling pathway. Furthermore, we found that coapplication of IMQ and ANO1 inhibitors on the ears of mice reduced not only symptoms of IMQ-induced psoriasis, but also expression of ANO1 and phosphorylation of ERK1/2. In addition, expression of inflammation-related cytokines including IL-17, IL-6, and TNF-α upregulated by application of IMQ was decreased by ANO1 inhibition, showing that psoriasis-like inflammation is alleviated by inhibition of ANO1. To our knowledge, this is the first study to identify the effects of ANO1 on proliferation of keratinocytes and the pathophysiology of psoriasis. These results not only aid in understanding the underlying mechanisms of ANO1 in epidermal keratinocyte hyperproliferation, but also suggest the potential of ANO1 as a treatment target of psoriasis.

## 4. Materials and Methods

### 4.1. Cell Culture

HaCaT cells were provided kindly by Prof. Sang-Jin Kang (CHA University, Seongnam, Korea). The cells were cultured in Dulbecco’s Modified Eagle’s Medium (DMEM; Thermo Fisher Scientific, Waltham, MA, USA) supplemented with 10% fetal bovine serum (FBS; Thermo Fisher Scientific, Waltham, MA, USA), 100 U/mL penicillin, and 100 µg/mL streptomycin. To restore the undifferentiated state, we grew HaCaT cells in keratinocyte serum-free medium (KSFM, 17005042; Thermo Fisher Scientific, Waltham, MA, USA) supplemented with bovine pituitary extract (BPE), 5 ng/mL EGF, 100 U/mL penicillin, and 100 µg/mL streptomycin for 2 weeks.

### 4.2. Human Tissue Samples

Normal and psoriatic tissues of patients with psoriasis were obtained from Bundang CHA Hospital (Seongnam, Korea). The study was approved by the Institutional Review Board of CHA University (approval number, CHAMC 2017-05-007-001); informed written consent was obtained from tissue donors. Clinical characteristics of donors were presented in Table S1.

### 4.3. Cell Proliferation Assay

Cell proliferation was assayed using the MTS assay kit (Promega, Madison, WI, USA) according to the manufacturer’s instructions. Prior to treatment with ANO1 blockers or siRNAs, HaCaT cells were seeded on 96-well microplates at a density of 5000 cells per well and incubated at 37 °C and 5% CO_2_ for 24 h. To evaluate the effects of ANO1 blockers on HaCaT cell proliferation, we applied 100 µL of fresh medium containing DMSO (vehicle) or ANO1 blockers (30 µM T16Ainh-A01 [Sigma-Aldrich, Steinheim, Germany] or 10 µM Ani9 [Sigma-Aldrich, Steinheim, Germany]) solved in DMSO to each well and incubated at 37 °C and 5% CO_2_ for 1, 2, 3, and 4 days. Cells were treated with vehicle or ANO1 blockers every day. For proliferation analysis, 20 µL of the mixed MTS/PMS solution was added to each well containing 100 µL culture medium each day, and the plate was incubated for 1 h at 37 °C and 5% CO_2_. Cell proliferation was quantified by measuring the absorbance at 490 nm with a SpectraMax i3x multi-microplate reader (Molecular Devices, San Jose, CA, USA).

### 4.4. Knock-Down of ANO1

Small interference RNAs (siRNAs) for ANO1 and negative controls were generated from Genolution (Seoul, Korea). The siRNA sequences for ANO1 are 5′-GAAGAUGUACCACAUUAAUUU-3′ and 5′-AUUAAUGUGGUACAUCUUCUU-3′. The siRNA sequences for the negative control are 5′-CCUCGUGCCGUUCCAU- CAGGUAGUU-3′ and 5′-CUACCUGAUGGAACGGCACGAGGUU-3′. HaCaT cells were seeded on a 6 well-plate and incubated at 37 °C and 5% CO_2_ to 50–60% confluence. The culture medium was replaced with BPE-supplemented KSFM contained 5 ng/mL EGF, and 50 pmol of siRNAs was transfected into the cells using Lipofectamine 3000 transfection reagent (Thermo Fisher Scientific, Waltham, MA, USA) according to the manufacturer’s instructions. The cells were incubated at 37 °C and 5% CO_2_ for 3 days.

### 4.5. Halide-Sensitive YFP Imaging

Fisher rat thyroid (FRT) cells that stably express YFP-H148Q/I152L/F46L and ANO1 were seeded on 96-well microplates at a density of 20,000 cells per well and incubated at 37 °C in a humidified atmosphere of 5% CO_2_ for 24 h. The cells were washed twice with 1X PBS and then incubated in 140 mM NaCl solution containing test compounds at 37 °C in a humidified atmosphere of 5% CO_2_ for 30 min. After incubation, to stimulate ANO1-mediated I^-^ influx, cells were injected with 140 mM NaI solution containing 100 μM ATP at 1 s, and fluorescence was measured every 0.2 s (total of 6 s). Binding of influxed I^−^ to YFP protein due to the test compounds induced reduction of YFP fluorescence and the signal of fluorescence was measured using a SpectraMax i3x multi-microplate reader (Molecular Devices, San Jose, CA, USA) that was equipped with 488 nm excitation and 520 nm emission filters.

### 4.6. Animals and IMQ-Induced Psoriasis Model Construction

Twenty-one 7-week-old female BALB/c mice (18–20 g per mouse) were purchased from Orient Bio Inc. (Seongnam, Korea) and underwent a 1-week period of acclimatization. The mice were housed on a 12-h:12-h light:dark cycle at room temperature (22 ± 1 °C) throughout the experiments. All experimental procedures were approved by the Institutional Animal Care and Use Committee (IACUC) of CHA University (IACUC170029). All experimental groups were housed with ad libitum access to food and water during the experiments. The mice were divided randomly into four groups as follows: control group (Vaseline-treated mice, *n* = 7), IMQ (3M Healthcare, Loughborough, UK) group (IMQ-treated group, *n* = 7), IMQ + 30 µM A01 group (IMQ- and 30 µM T16Ainh-A01-cotreated group, *n* = 7), and IMQ + 100 µM A01 group (IMQ- and 100 µM T16Ainh-A01-cotreated group, *n* = 7). 

Mice were treated with a daily dose of 25 mg of 5% IMQ cream on the dorsal and ventral aspects of the right ear skin once a day for 7 consecutive days. The control group received a similar dose of Vaseline cream on their right ears. IMQ + 30 µM A01 and IMQ + 100 µM A01 groups received 15 µL of 30 µM and 100 µM T16Ainh-A01, respectively, on their right ears at 30 min before IMQ treatment and then received additionally 25 mg of 5% IMQ cream, respectively. Control and IMQ groups also received 15 µl of DMSO at 30 min before Vaseline and IMQ treatments, respectively. 

### 4.7. Scoring Severity of Skin Inflammation

Ear thickness was measured using a micrometer (Mitutoyo, Tokyo, Japan) just before applying the creams and then every day for 7 consecutive days. The severity of inflammation of the ear skin was scored every day for 7 consecutive days based on the clinical Psoriasis Area and Severity Index (PASI) as reported previously [43,62]. Thickening, erythema, and scaling were scored independently from 0 to 4 depending on severity: none, 0; slight, 1; moderate, 2; marked, 3; very marked, 4. Scoring of erythema severity was performed using a scoring table with red taints. The severity of inflammation was represented by the cumulative score (thickening plus erythema plus scaling) (scale 0–12).

### 4.8. RT-PCR

Total RNA was extracted from HaCaT cells and mouse right ears (*n* = 7 per group) using TRIzol reagent (Thermo Fisher Scientific, Waltham, MA, USA) and reverse-transcribed with the TOPscript^TM^ cDNA Synthesis kit (Enzynomics, Seoul, Korea). The synthesized cDNA was mixed with Dyne 2X PreMIX-HOT (DYNE Bio, Seongnam, Korea) and gene-specific primers. The amplification conditions were as follows: initial melt at 95 °C for 3 min, followed by 22 to 35 cycles of denaturation at 95 °C for 30 s, annealing at 58–60 °C for 45 s, extension at 72 °C for 45 s, and final extension at 72 °C for 10 min. The amplified products were evaluated by 1% agarose gel electrophoresis. The density of PCR products obtained from mouse ears was measured using ImageJ software (v 1.50i, National Institutes of Health, Bethesda, MD, USA). The primers used for amplification of candidate genes are presented in Table S2.

### 4.9. Western Blot Analysis

Mouse-ear tissues prior to lysis were disrupted using liquid nitrogen. HaCaT cells and the disrupted mouse ear tissues were lysed with RIPA buffer (Bio-sesang, Seongnam, Korea) containing protease and phosphatase inhibitor (Roche, Mannheim, Germany) and incubated at 4 °C for 30 and 60 min, respectively. The cell and tissue lysates were centrifuged at 15,000 rpm and 4 °C for 30 min, and the supernatants were transferred into fresh tubes. The extracted protein concentration was measured using the Bio-Rad protein assay kit (Bio-Rad, Hercules, CA, USA) according to the manufacturer’s instructions. The proteins were denatured by heating, separated by SDS-PAGE, and transferred to PVDF membranes (Millipore, Billerica, MA, USA). The membranes were blocked with TBS-T buffer containing 5% non-fat skim milk at room temperature for 1 h. The membranes were incubated with primary antibodies overnight at 4 °C: anti-ANO1 (1:2000) (Ab64085, Abcam, Cambridge, MA, USA), anti-β-actin (1:5000) (sc-47778, Santa Cruz Biotechnology, Santa Cruz, CA, USA), anti-GAPDH (1:5000) (sc-25778, Santa Cruz Biotechnology, Santa Cruz, CA, USA), anti-phospho-ERK1/2 (anti-pERK1/2; 1:1000) (#9106, Cell Signaling Technology, Berkeley, CA, USA), anti-ERK1/2 (1:1000) (#9102, Cell Signaling Technology, Berkeley, CA, USA), anti-phospho-AKT (anti-pAKT; 1:1000) (#4060, Cell Signaling Technology, Berkeley, CA, USA), and anti-AKT (1:1000) (#9272, Cell Signaling Technology, Berkeley, CA, USA). After washing the membranes with TBS-T buffer, they were incubated with HRP-conjugated secondary antibodies at room temperature for 1 h. The membrane-bound proteins were visualized using SuperSignal West Pico PLUS Chemiluminescent Substrate (Thermo Fisher Scientific, Waltham, MA, USA). 

### 4.10. Immunocytochemistry and Immunohistochemistry

For immunocytochemistry (ICC), HaCaT cells were seeded on coverslips and incubated at 37 °C and 5% CO_2_ for 24 h. The coverslips were washed with 1X PBS three times and fixed with 4% paraformaldehyde at room temperature for 15 min. The cells on coverslips were permeabilized with 0.5% Triton X-100 for 7 min and incubated in a blocking solution containing 0.5% BSA and 0.05% Triton X-100 for 1 h at room temperature. The coverslips were incubated in a blocking solution containing anti-ANO1 (1:1,500) (14476S, Cell Signaling Technology, Danvers, MA, USA) overnight at 4 °C. The coverslips were incubated in a blocking solution containing goat IgG (H + L) cross-adsorbed secondary antibody (1:500) (A-11078, Thermo Fisher Scientific, Waltham, MA, USA) for 1 h at room temperature, and then the nuclei were stained with Hoechst 33,342 (1:500) (H1399, Thermo Fisher Scientific, Waltham, MA, USA) for 5 min at room temperature. The coverslips were mounted with VECTASHIELD mounting medium (Vector Laboratories, Burlingame, CA, USA). The cell images were recorded using a Zeiss LSM 700 confocal laser scanning microscope (Zeiss LSM 700, Carl Zeiss, Jena, Germany) with DAPI and AF488 excitation filters. 

For immunohistochemistry (IHC), human skin and mouse ear tissues were fixed with 4% formaldehyde for 24 h, washed with distilled water, dehydrated gradually with a series of 70–100% ethanol washes, immersed in xylene, and embedded in paraffin. The paraffin-embedded psoriatic patient and mouse-ear tissues sliced into 5-μm sections were transferred onto slides, and the slides were deparaffinized with xylene. The slides were hydrated through washes with graded alcohols and water. For hematoxylin and eosin (H&E) staining, the slides containing mouse right ear tissue sections were H&E stained, dehydrated, and mounted. For antigen retrieval, the slides were heated at 95 °C for 20 min in 10 mM sodium citrate buffer at pH 6.0. After cooling, the slides were quenched with 3% H_2_O_2_ for 20 min. The slides were incubated with a rabbit anti-ANO1 antibody (1:100) (ab64085, Abcam, Cambridge, MA, USA) overnight at room temperature and were incubated with HRP-conjugated secondary antibody for 1 h at room temperature. The tissues were developed on the slides using 3,3′-diaminobenzidine tetrahydrochloride (DAB) (Sigma-Aldrich, St. Louis, MO, USA). Images were captured using a Leica ICC50E microscope (Leica, Wetzlar, Germany) or an Axio Scan.Z1 slide scanner (Carl Zeiss Microscopy GmbH, Jena, Germany). 

Digital image of each slide was analyzed using the Zen lite software (Blue Edition, Carl Zeiss, Oberkocken, Germany). The signal intensity of ANO1 per ten cells in the epidermis of human tissues or mouse ears was measured using Zen 3.1 software (Blue Edition, Carl Zeiss, Oberkocken, Germany). ANO1 intensity of ten cells per unit area was obtained from 3 samples per group in human tissues and mouse ears.

### 4.11. Statistical Analysis

Statistical analyses were conducted with GraphPad Prism Prism 8 software (San Diego, CA, USA). Comparison of *ANO1* mRNA expression in human normal and psoriasis skin tissues was performed using Student’s *t*-test. Comparisons of proliferation ratios, protein expression levels, ear thicknesses, and mRNA expression levels among control and test groups were performed using one-way ANOVA followed by Tukey’s multiple comparison test or two-way ANOVA followed by Bonferroni’s post-hoc test. All data are expressed as the mean ± standard error of the mean (SEM). *p* < 0.05 was considered statistically significant.

## Data Availability

The datasets generated and analyzed in this study are available from the corresponding author upon reasonable request.

## Supplementary Materials

The following are available online at https://www.mdpi.com/article/10.3390/ijms22137145/s1.

## Abbreviations

ANO1	Anoctamin1
BPE	Bovine pituitary extract
BPH	Benign prostatic hyperplasia
DAB	3,3′-diaminobenzidine tetrahydrochloride
DCs	Dendritic cells
DIDS	4,4-diisothiocyano-2,2-stilbenedisulfonic acid
DMEM	Dulbecco’s Modified Eagle’s Medium
FBS	Fetal bovine serum
FRT	Fisher rat thyroid
H&E	Hematoxylin and eosin
HNSCC	Head and neck squamous cell carcinoma
ICC	Immunocytochemistry
IHC	Immunohistochemistry
IMQ	Imiquimod
KSFM	Keratinocyte serum free medium
MDCs	Myeloid DCs
NPPB	5-nitro-2-(3-phenylpropylamino)-benzoic acid
PASI	Psoriasis area and severity index
PDCs	Plasmacytoid DCs
SEM	Standard error of the mean
si-ANO1	siRNA targeting *ANO1*
siRNAs	Small interference RNAs
Th	T-helper
TMEM16A	Transmembrane protein 16A
TNF-α	Tumor necrosis factor-α
