# Supplementary material for: Anoctamin1 Induces Hyperproliferation of HaCaT Keratinocytes and Triggers Imiquimod-Induced Psoriasis-Like Skin Injury in Mice
Source: Int J Mol Sci. 2021 Jul 1;22(13):7145. doi: 10.3390/ijms22137145 (PMC8268182; doi:10.3390/ijms22137145)
Supplement: Supplementary file 1 [file ijms-22-07145-s001.zip › ijms-1220059-supplementary.pdf]

Quantitative reverse transcription PCR (qRT-PCR)

To validate *ANO1* mRNA expression, we obtained normal (n = 3) and psoriatic (n = 3) tissues of patients with psoriasis from Bundang CHA Hospital (Seongnam, Korea). The study was approved by the Institutional Review Board of CHA University (approval number, CHAMC 2017-05-007-001). Total RNA was extracted using Trizol reagent (Thermo Fisher Scientific, Waltham, MA, USA) and reverse-transcribed with the TOPscript^{TM} cDNA Synthesis kit (Enzynomics, Seoul, Korea). For quantitative PCR, 5 μl of TB Green Premix Ex Taq (Takara Bio, Otsu, Japan), 2 pmol of forward primer, 2 pmol of reverse primer, and 1 μl of cDNA were added with water to a final volume of 10 μl. The mixture was amplified for 40 cycles with a ViiA7 real-time PCR system (Thermo Fisher Scientific, Waltham, MA, USA). The cycle number at which a statistically significant increase in each gene was first detected (threshold cycle, Ct) was then normalized to the Ct for *GAPDH*. The relative expression differences of *ANO1* between normal and psoriasis were calculated using the $2^{-\Delta\Delta CT}$ method [1]. The primers used for the amplification of *ANO1* and *GAPDH* are as follows: *ANO1* (forward), 5'-CAT CAG CCA GCA GAT CCA CA-3'; *ANO1* (reverse), 5'-CAG GCT TTG GTG TTG TGG TG-3'; *GAPDH* (forward), 5'-ACC CAG AAG ACT GTG GAT GG-3'; *GAPDH* (reverse), 5'- TTC TAG ACG GCA GGT CAG GT-3'.

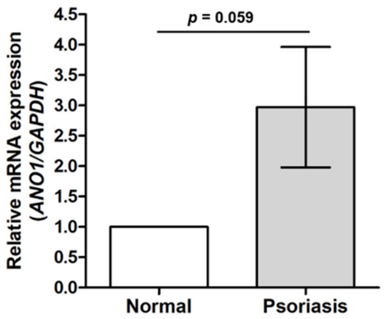

Supplementary Figure S1. Comparison of *ANO1* mRNA expression in human normal and psoriasis tissues. Total RNA was extracted from normal (n = 3) and psoriasis (n = 3) skin tissues and ANO1 mRNA expression was analyzed by qRT-PCR. Values represent the mean ± SEM (n = 3). Statistical analysis was performed using Student's *t*-test. $P < 0.05$ was considered statistically significant.

Reference

1. Choi, M.R.; Jung, K.H.; Park, J.H.; Das, N.D.; Chung, M.K.; Choi, I.G.; Lee, B.C.; Park, K.S.; Chai, Y.G. Ethanol-induced small heat shock protein genes in the differentiation of mouse embryonic neural stem cells. *Arch. Toxicol.* **2011**, 85, 293-304.

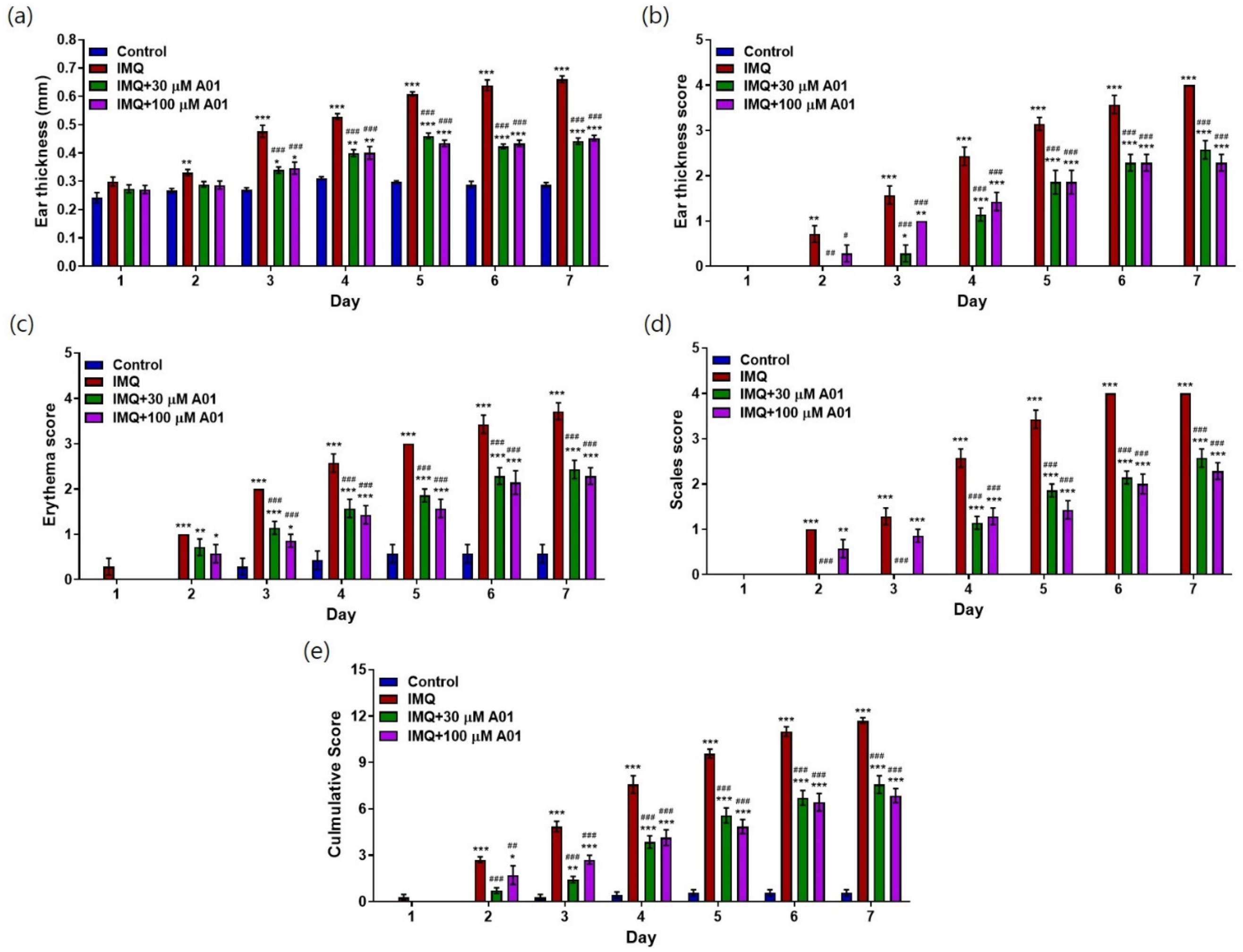

Supplementary Figure S2. Significant changes of IMQ-induced psoriasis-like symptoms in mouse ears by inhibiting ANO1. Significant differences of makers of psoriasis-like symptoms among four groups that were described in Figure 4 of the main manuscript were analyzed using two-way ANOVA and Tukey's post-hoc tests. *: significantly different from control group per day (*$p < 0.05$, **$p < 0.01$, and ***$p < 0.001$). $^{\#}$: significantly different from IMQ group per day ($^{\#}p < 0.05$, $^{\#\#}p < 0.01$, and $^{\#\#\#}p < 0.001$). Imiquimod, IMQ; T16Ainh-A01, A01.

Supplementary Table S1. Clinical characteristics of donors included in this study

Donor	Sex	Age (year)	BSA (m^2)	PASI		Experiment
				Normal	Psoriasis	
1	M	48	2	ND	2.1	IHC
2	M	42	8	ND	8.3	IHC
3	F	32	15	ND	15.2	IHC
4	M	46	15	ND	15	qRT-PCR
5	M	31	10	ND	10	qRT-PCR
6	M	39	5	ND	5.6	qRT-PCR

Both normal (healthy) and psoriasis skin tissues per donor were biopsied. Psoriasis skin was only evaluated by the standard of PASI. BSA, body surface area; F, female; IHC, immunohistochemistry; M, male; ND, not determined; PASI, psoriasis area and severity index.

Supplementary Table S2. Primers used in RT-PCR

Species	Gene	Forward (5′-3′)	Reverse (5′-3′)	Annealing temperature (°C)
Human/mouse	*ANO1*	CTTCAAAGGCCGGTTTGTTGG	TTGACGAAGCCGTGCATGGTC	59
Mouse	*IL-17A*	CAAACACTGAGGCCAAGGAC	TCTTCATTGCGGTGGAGAGT	59
Mouse	*IL-17F*	ATTCTGAGGGAGGTAGCAGC	TGGAATTCACGTGGGACAGA	58
Mouse	*IL-22*	GACAGGTTCCAGCCCTACAT	GTTCCCCAATCGCCTTGATC	59
Mouse	*IL-23*	GCCTAGGAGTAGCAGTCCTG	TCCTTGAGTCCTTGTGGGTC	58
Mouse	*IL-6*	CACTCCCAACAGACCTGTCT	TGCAAGTGCATCATCGTTGT	58
Mouse	*IL-1b*	ACTCATTGTGGCTGTGGAGA	AGCCTGTAGTGCAGTTGTCT	58
Mouse	*TNF*	CCTCCTCTCTGCCGTCAAGA	AAGTAGACCTGCCCGGACTC	60
Human	*GAPDH*	TTTGGTCGTATTGGGCGCCTG	CCATGACGAACATGGGGGCAT	59
Mouse	*GAPDH*	GTGAAGGTCGGTGTGAACGGA	CCCATCACAAACATGGGGGCA	59
